# Catalytic Features and Thermal Adaptation Mechanisms of a Deep Sea Bacterial Cutinase-Type Poly(Ethylene Terephthalate) Hydrolase

**DOI:** 10.3389/fbioe.2022.865787

**Published:** 2022-04-26

**Authors:** Yu Liu, Chen Liu, Huan Liu, Qi Zeng, Xinpeng Tian, Lijuan Long, Jian Yang

**Affiliations:** ^1^ CAS Key Laboratory of Tropical Marine Bio-resources and Ecology, Guangdong Key Laboratory of Marine Materia Medica, South China Sea Institute of Oceanology, Chinese Academy of Sciences, Guangzhou, China; ^2^ University of the Chinese Academy of Sciences, Beijing, China; ^3^ Southern Marine Science and Engineering Guangdong Laboratory (Guangzhou), Guangzhou, China; ^4^ Guangzhou Quality Supervision and Testing Institute, Guangzhou, China

**Keywords:** Poly (ethylene terephthalate), cutinase, product inhibition, biorecycling, molecular dynamic simulation, protein engineering

## Abstract

Poly (ethylene terephthalate) (PET) plastic is chemically inert and persistent. Massive quantities of PET waste end up in landfill sites and oceans, posing major global pollution concerns. PET degrading enzymes with high efficiency provide plastic recycling and bioremediation possibilities. Here, we report a novel cutinase, *Mt*Cut with distinct catalytic behaviors, derived from the deep sea *Nocardiopsaceae* family strain. Biochemical analyses showed *Mt*Cut efficiently hydrolyzed PET at ambient temperatures and in an exo-type manner. The activity and stability of *Mt*Cut were enhanced by the addition of calcium ions. Notably, no hydrolysis products inhibition was observed during PET depolymerization, suggesting *Mt*Cut is a better biocatalyst when compared to other PET hydrolases. In addition, structural components associated with thermal adaptation were investigated using molecular dynamic (MD) simulations, and key regions regulating *Mt*Cut thermostability were identified. Our biochemical and structural analyses of *Mt*Cut deepen the understanding of PET hydrolysis by cutinases, and provide invaluable insights on improvement and performance engineering strategies for PET-degrading biocatalysts.

## Introduction

Since the 1950s, plastic materials have become essential in modern society and have greatly changed human life-styles (Andrady and Neal, 2009). Huge market demands and wide-spread plastics use have meant that the cumulative plastics output would reach 12,000 million metric tons by 2050 (Geyer et al., 2017). Inappropriate disposal and chemically recalcitrant properties render these plastics as bulk environmental contaminants, accounting for more than 10% of municipal solid waste (Jambeck et al., 2015). Thus, plastic contamination of the natural environment is a grave concern. Critical issues generated by plastic pollution are the formation micro/nanoplastics which circulate *via* food chains and absorb toxic compounds threatening human and animal health (Amaral-Zettler et al., 2020; Sun et al., 2020). The only way to permanently eliminate plastic waste before it enters ecosystems is *via* destructive thermal treatment, such as combustion or pyrolysis; however, these processes induce other environmental problems (Simoneit et al., 2005; Al-Salem et al., 2017). Therefore, plastic biorecycling, aimed at material recovery, is highly important for resource saving and improving eco-friendly processes (Wei et al., 2020).

Polyethylene terephthalate (PET) is a low weight, versatile, and durable synthetic aromatic polyester used to produce textile fibers and resins for single-use packaging and beverage bottles (Awaja and Pavel, 2005; Welle, 2011). PET has a readily biodegradable structure comprising repeated ester bonds of terephthalate and ethylene glycol (Kawai, 2021). However, crystallinity, high molecular weight, and extremely hydrophobic surface of PET are barriers to effective biorecycling (Samak et al., 2020). In 2016, a newly discovered bacterium, *Ideonella sakaienesis* 201-F6, reportly used PET as a major carbon and energy source for growth (Yoshida et al., 2016). The PET depolymerase isolated from *I. sakaienesis* 201-F6 (*Is*PETase, EC 3.1.1.101) hydrolyzed PET to momo (2-hydroxyethyl) terephthalic acid (MHET), with trace quantities of bis (2-hydroxyethyl) terephthalic acid (BHET), and terephthalic acid (TPA). However, several cutinases (EC 3.1.1.74) were previously reported to depolymerize PET before *Is*PETase (Chen et al., 2020; Arnling Baath et al., 2022). Importantly, *Is*PETase is highly homologous to bacterial cutinases.

Cutinases are a group of small serine esterases of the α/β hydrolase-fold family and are secreted by plant pathogens to attack and degrade hydrophobic apoplastic barriers comprising the polyesters, cutin and suberin (Nawrath, 2002; Chen et al., 2013; Chen et al., 2020). The enzymes possess a Ser-His-Asp catalytic triad and an oxyanion hole for catalyzing the hydrolysis of hydrophobic compounds (Bauer et al., 2020). Unlike lipases, cutinases active sites are located in a shallow binding cleft, without an amphipathic loop (Longhi and Cambillau, 1999). Due to this architecture, cutinases hydrolyze high molecular weight polymer chemicals such as PET (Arnling Baath et al., 2022). The first report on PET enzymatic hydrolysis involved a cutinase derived from *Thermobifida fusca* (Müller et al., 2005). Since then, various cutinase homologs from actinomycetes, especially the family *Nocardiopsaceae*, have been discovered as promising PET hydrolysis candidates (Thumarat et al., 2012; Kawai et al., 2014). PET hydrolysis appears to be a promiscuous function of cutinases, since a commercialized fungal cutinase (HiC) from *Humicola insolens*, with no homology to bacterial cutinases, also exhibited high activity against PET (Ronkvist et al., 2009).

Protein engineering can improve and tailor cutinase properties. An engineered PET hydrolase, ICCG with four mutations on the leaf-branch compost cutinase (LCC), displayed simultaneous improvements in activity and stability toward efficient depolymerization, outperforming all PET hydrolases so far (Tournier et al., 2020). Despite these breakthroughs, it is noteworthy that all known PET hydrolases exhibit low turnover rates, which render efficient PET bioremediation largely impossible. Based on the huge genetic diversity in nature, it is entirely conceivable that more PET depolymerases with excellent performances remain to be discovered.

In this work, we describe the biochemical characteristics of a novel cutinase (*Mt*Cut), with PET hydrolyzing activity, from *Marinactinospora thermotolerans* DSM45154, a deep sea (−3,865 m) strain of the *Nocardiopsaceae* family (Tian et al., 2009). The enzyme efficiently transforms PET into MHET and TPA at ambient temperatures, with no significant inhibitory effects from hydrolysis products. We also focused on the distinct temperature adaptations of *Mt*Cut and its thermophilic counterpart, ICCG using structural comparisons, and identified key regions closely associated with enzyme thermostability. Based on these analyses, our findings provide invaluable insights on the catalysis and thermostability of cutinase-like PET hydrolases.

## Materials and Methods

### Protein Expression and Purification

A 786 base pair (bp) gene fragment encoding *Mt*Cut (GenBank accession: SJZ42839) without the N-terminal signal peptide was amplified from genomic DNA of *M. thermotolerans* DSM45154. Using seamless cloning (Transgen Biotech, Beijing, China), the gene fragment was cloned into the pET22b (+) vector (Novagen, Madison, WI, United States) between the *Nde*I and *Xho*I restriction sites, along with a C-terminal hexahistidine (6 × His) tag. The construct was verified by DNA sequencing. Recombinant protein was produced in *Escherichia coli* BL21 (DE3) (Novagen) grown in Luria Broth media containing 100 μg/ml ampicillin at 37°C until the optical density reached 0.6–0.8 at 600 nm. Protein expression was then induced by adding 0.5 mM isopropyl β-d-1-thiogalactopyranoside (Sangon, Shanghai, China) at 16°C for 16 h. Cells were harvested by centrifugation at 5,000 × *g* for 10 min, and then the pellet resuspended in 20 mM Tris-HCl (pH 8.0), 500 mM NaCl, and 5 mM imidazole. Cells were disrupted by sonication on ice, and cell debris removed by centrifugation at 10,000 × *g* for 30 min. The clarified lysate was loaded onto equilibrated Ni-NTA resin (Qiagen, Hilden, Germany). After rinsing in binding buffer (20 mM Tris-HCl (pH8.0), 500 mM NaCl, 5 mM imidazole) and washing buffer [20 mM Tris-HCl (pH8.0), 500 mM NaCl, 20 mM imidazole], recombinant protein was eluted using 20 mM Tris-HCl (pH8.0), 200 mM imidazole. The proteins were further purified by AKTA primer plus system with a HiPrep DEAE FF 16/10 column (GE Healthcare, Chicago, United States) over a linear 0–1.0 M NaCl gradient. Purified proteins were concentrated using an Amicon-Ultra-15 device (Molecular Weight Cut-off = 10 kDa, Merck-Millipore Co., United States). Protein purity was analyzed by sodium dodecyl sulfate-polyacrylamide gel electrophoresis and protein concentrations determined by absorbance at 280 nm using the molar extinction coefficient calculated from amino acid sequence composition (https://web.expasy.org/protparam/).

### Cutinase Assay

All reactions were performed in 96-well plates in 200 μl total volume. We tested *para-*nitrophenyl (*p*NP) esters (Aladdin, Shanghai, China) with chain lengths of C_2_, C_4_, C_6_, C_8_, C_10_, C_12_, C_14_, C_16_, and C_18_ as cutinase type substrates. To 190 μl 20 mM Tris-HCl (pH8.0) buffer plus dissolved *p*NP esters, we added 10 μl purified *Mt*Cut (5 μg/ml). Reaction times ranged from 10–30 min at 30°C, and plates were measured in triplicate. The *p*-nitrophenol product was measured at 405 nm using an EnSight™ Multimode microplate reader (PerkinElmer Inc., Spokane, WA, United States). One unit of enzyme activity was defined as the amount of enzyme required to convert 1.0 μmol *p*-nitrophenol per min under standard conditions. Data, *v*_o_ (mM/min) versus [*S*]_0_ (mM) were fitted to a Michaelis-Menten model to calculate kinetic parameters. To determine the optimal temperature and pH, reaction samples using *p-*NP butyrate as substrate were incubated in the temperature range 5–70°C, and pH range 7.0–9.5, respectively. Enzyme thermal and pH stability parameters were measured after a specified incubation period at a constant temperature and pH range. Residual activity was determined after 10 min incubation at 30°C. The effects of calcium ions and EDTA on enzyme properties were determined by adding calcium ions or EDTA at a final concentration of 1 mM. We measured the effect of final calcium ion concentration ranged from 0 to 500 mM on the cutinase activity.

### PET Crystallinity Analysis

We estimated the thermal characteristics and crystallinity of PET using differential scanning calorimetry (Netzsch DSC 214, Bavaria, Germany) with a heating rate of 10°C/min in a nitrogen environment. Crystallinity (*θ*) calculations were based on the following equation (Tournier et al., 2020):

$$\theta =\frac{\left(\Delta H_{f}-\Delta Hcc\right)}{\Delta H_{f}100\%}\times 100\%$$

Where Δ*H*_f_ is the melting enthalpy (J/g), Δ*H*_cc_ is the enthalpy of cold crystallization (J/g), Δ*H*_f_ 100% is the theoretical melting enthalpy of 100% PET crystallization with a value of 140.1 J/g. PET microparticle (KAI YUAN Plastication Technology Co., Dongguan, China) has a melting temperature (*T*_m_) of 247.2°C, a crystallization temperature of 173.7°C, a glass transition temperature (*T*_g_) of 79.1°C, a melting enthalpy (Δ*H*_f_) of 59.78 J/g, and with no observed enthalpy of cold crystallization (Δ*H*_cc_), thus the crystallinity percentage is 42.67%. PET low crystallinity (lc-PET) films (Goodfellow Ltd., Bad Nauheim, Germany) have a *T*_m_ of 247.5°C, a crystallization temperature of 173.8°C, a *T*_g_ at of 71.9°C, a Δ*H*_f_ of 37.86 J/g and a Δ*H*_cc_ of 23.4 J/g, thus the crystallinity percentage is 10.32%.

### PETase Assay

PET-hydrolytic activity was measured as previously described (Espino-Rammer et al., 2013; Chen et al., 2021; Kaabel et al., 2021). Briefly, 10 μg/ml purified enzyme was incubated with 4 mg high-crystallinity PET microparticles (42.67% crystallinity) in 20 mM Tris-HCl (pH 8.5), 500 mM NaCl, 10% (*v*/*v*) dimethyl sulfoxide, and 10 mM CaCl_2_. Samples were incubated at 150 rpm, 40°C. The reaction was terminated by adding 10 μl 1 M HCl to a 2 ml reaction supernatant, and a 25 μl aliquot analyzed by high performance liquid chromatography (Agilent 1,200, CA, United States) equipped with an SB C-18 column (5 μm, 4.6 × 150 mm, Agilent). The mobile phase was 20 mM phosphoric acid containing a 10–100% methanol linear gradient flowing at 1 ml/min. Eluates were monitored at 240 nm and peak areas for BHET, MHET, and TPA determined based on known standards.

### BHETase and MHETase Assay

BHETase and MHETase assays were performed in a total 1,000 μl volume with BHET or MHET dissolved in 900 μl 20 mM Tris-HCl (pH8.0). The concentration gradient of MHET and BHET was stetted as a range from 0.05 to 1.0 mM. Enzymatic reaction was started by adding 100 ul of enzyme solution at 40°C in triplicate. The reaction time of *Mt*Cut (0.02 mg/ml), ICCG (0.05 mg/ml), and *Is*PETase (0.05 mg/ml) were 15, 120, and 120 min, respectively. Substrate reduction during the reaction was detected by high performance liquid chromatography. One unit of enzyme activity was defined as the amount of enzyme required to reduce 1.0 μmol substrate per min under standard conditions. Data, *v*_o_ (mM/min) versus [*S*]_0_ (mM) were fitted to a Michaelis-Menten model to calculate kinetic parameters.

### Site-Directed Mutagenesis

Primers (Supplementary Table S1) were designed with substituted codons at target sites to generate mutants using one-step site-directed mutagenesis (Zheng et al., 2004). PCR was performed using Phanta Master DNA polymerase (Vazyme, Nanjing, China) with the following parameters: 95°C for 10 min, followed by 32 cycles of 95°C for 30 s, 50°C for 30 s, and 72°C for 4 min and then 72°C for 10 min. PCR products were digested with *Dpn*I (MBI Fermentas, Vilnius, Lithuania) to remove the methylated parent plasmid and then purified using a PCR purification kit (GenStar, Beijing, China). The linearized plasmid derivatives were transformed into *E. coli* XL1-Blue competent cells to derive mutant plasmids. After purification and identification, target plasmids were transformed into *E. coli* BL21 (DE3) cells for mutant enzymes production, and protein was expressed and purified as described above.

### Differential Scanning Fluorimetry

DSF studies were performed to assess the thermal stability of *Mt*Cut and ICCG by determining *T*_m_ values. White clear 96-well PCR plates were used, with wells containing 12.5 μl buffer A (20 mM Tris-HCl, pH8.0, 200 mM NaCl), 10 μl 1 mg/ml protein solution in buffer A, and 2.5 μl 50 × SYPRO Orange (Sigma-Aldrich, St Louis, United States) solution in ddH_2_O, to a final volume of 25 μl. DSF studies were conducted using a Bio-Rad CFX96 real-time PCR system (Bio-Rad, Hercules, CA, United States), set on the fluorescence resonance energy transfer channel using 490 nm excitation and 580 nm emission filters. Samples were heated from 10 to 95°C at 0.05°C/s. Protein unfolding was monitored by detecting changes in SYPRO Orange fluorescence. *T*_m_ values were determined from the peaks of the first derivatives of the melting curve using CFX Manager software (Bio-Rad).

### Molecular Docking

The *Mt*Cut protein structure was predicted using the ColabFold implementation of AlphaFold2 with default parameters (Jumper et al., 2021; Mirdita et al., 2021). The built *Mt*Cut structure was prepared for computational docking using the protein preparation wizard in MOE software (version 2019.0102, Chemical Computing Group, Montreal, Canada). Hydrogen atoms were added to the protein–ligand complex at pH 7. The hydrogen bond network and protein structure were further optimized to the overall lowest potential energy configuration using protonate 3D. The ligands were docked using rigid-fit method with the carbonyl oxygen of the ester bond constrained in the oxyanion hole formed by Ser178 and His 256). The top-ranked docking conformations based on the default scoring function of GOLD were selected for further investigation.

### Molecular Dynamics Simulations

All MD simulations were performed using AMBER20 (Case et al., 2021). The AMBER FF19SB force field was applied and the SHAKE algorithm used to restrict all covalent bonds involving hydrogen atoms, with a time step of 2fs. The Particle Mesh Ewald method was used to treat long-range electrostatic interactions. For the solvated system, two steps minimization were performed before the heating step. The first 4,000 cycles of minimization were performed with all heavy atoms restrained with 50 kcal/(mol·Å2), whereas solvent molecules and hydrogen atoms were free to move. Then, non-restrained minimization was conducted using 2,000 cycles of steepest descent minimization and 2,000 cycles of conjugated gradient minimization. Afterwards, the whole system was heated from 0 to 308 K in 50 ps using Langevin dynamics at a constant volume, and then equilibrated for 400 ps at a constant pressure of 1 atm. A weak constraint of 10 kcal/(mol·Å2) was used to restrain all heavy atoms during heating steps. Periodic boundary dynamic simulations were conducted for the whole system using a constant composition, pressure, and temperature ensemble at a constant pressure of 1 atm and 308 K in the production step. In the production phase, a 100 ns simulation was conducted, after which the whole system was heated from 308 to 343 K in 100 ns using Langevin dynamics at a constant volume. Finally, 100 ns MD simulations were conducted at a constant pressure of 1 atm and 343 K. Trajectories were further analyzed using Cpptraj (Roe and Cheatham, 2013).

## Results

### Cutinase Activity of *Mt*Cut

**FIGURE 1:**
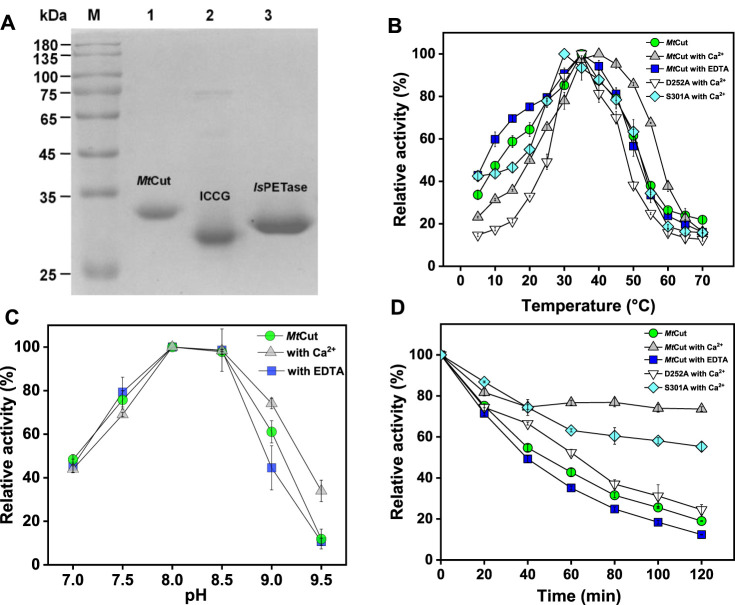
Cutinase activity of *Mt*Cut. **(A)** *Mt*Cut, ICCG, and *Is*PETase protein purity. The impact of temperature **(B)** and pH **(C)** on cutinase activity of *Mt*Cut and its mutants. **(D)** Thermal inactivation profiles of *Mt*Cut and its mutants in the absence/presence of 1 mM calcium ions or EDTA at 30°C. All the tests used *p*NP butyrate as substrate.

The amino acid sequence of the *Mt*Cut catalytic domain from *M. theromotolerans* DSM45154 shared 66% amino acid sequence identity with Cut190 from *Saccharomonospora viridis* (Kawai et al., 2014), 62% with Est119 from *Thermobifida alba* (Thumarat et al., 2012), 61% with TfCut1 from *Thermobifida fusca*, 57% with LCC from leaf-branch uncultured bacterium (Sulaiman et al., 2012), and 46% with *Is*PETase from *I. sakaiensis* (Han et al., 2017). These homologous enzymes are cutinase-like, with reported PET hydrolyzing activity. *Mt*Cut contained a conserved pentapeptide sequence motif (GHSMG), and a catalytic triad (Ser178-Asp224-His256) (Supplementary Figure S1). To examine *Mt*Cut catalytic function, a recombinant enzyme produced in *E. coli* was purified to homogeneity using a combined His-tag affinity and ion exchange chromatography approach (Figure 1A). The purified *Mt*Cut molecular mass agreed with the calculated 34 kDa value. Using *p*NP-C_4_ as the cutinase model substrate, *Mt*Cut exhibited its highest activity at 35°C and pH8.0 (Figures 1B,C). *Mt*Cut also showed typical cold-adapting properties, with more than 30 and 50% of full activity retained at 5 and 15°C, respectively. In Addition, the enzyme was unstable under heating treatment, with only 20% activity detected at 30°C for 2 h (Figure 1D). Enzyme kinetics toward *p*NP acyl esters of different chain lengths were determined under standard conditions (Table 1). Purified *Mt*Cut was active toward all tested *p*NP esters and generated the highest *k*_cat_ value against *p*NP-C_8_. Thus, *Mt*Cut was a cutinase-type enzyme rather than an esterase or true lipase. However, the affinity toward longer acyl chains (C_14_-C_18_) was higher than shorter chains (C_4_-C_12_). Overall, no significant difference in catalytic efficiency (*k*_cat_/*K*_m_) was found among C_2_-C_6_, C_14_-C_18_ substrates, respectively. Molecular docking was performed to analyze the underlying mechanism for different kinetic parameters. Interestingly, a good correlation between catalytic efficiency and ligand binding energy against *Mt*Cut was found (Supplementary Figure S2A). Closer examination of the docked structure does not show significant pose differences (Supplementary Figure S2B).

**TABLE 1:** *MtCut* kinetic parameters toward *p*NP esters^a^.

Substrate	*K*_m_ (mM)	*k*_cat_ (s^−1^)	*k*_cat_/*K*_m_ (M^−1^s^−1^)
*p*NP-C_2_	0.55 ± 0.04	4.47 ± 0.28	(8.19 ± 0.34) ×10^3^
*p*NP-C_4_	1.33 ± 0.15	14.10 ± 1.51	(1.06 ± 0.01) ×10^4^
*p*NP-C_6_	4.61 ± 1.04	41.72 ± 6.35	(9.23 ± 0.90) ×10^3^
*p*NP-C_8_	2.55 ± 0.28	79.39 ± 8.75	(3.11 ± 0.005) ×10^4^
*p*NP-C_10_	4.93 ± 0.53	69.85 ± 5.15	(1.42 ± 0.05) ×10^4^
*p*NP-C_12_	1.68 ± 0.12	30.52 ± 3.63	(1.81 ± 0.12) ×10^4^
*p*NP-C_14_	0.19 ± 0.04	9.87 ± 1.52	(5.14 ± 0.29) ×10^4^
*p*NP-C_16_	0.15 ± 0.01	4.79 ± 0.12	(3.25 ± 0.10) ×10^4^
*p*NP-C_18_	0.45 ± 0.02	14.31 ± 0.58	(3.18 ± 0.02) ×10^4^

a Data represent average values and triplicate experiments under standard conditions. The carbon subscript indicates of *p*NP, ester chain length.

### Enzyme Stability and Activity Are Enhanced by Calcium Ions (Ca^2+^)

**FIGURE 2:**
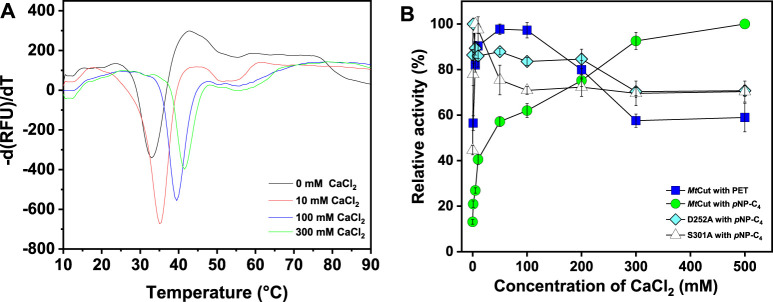
The effects of Ca^2+^ on *Mt*Cut thermostability and catalytic activity. **(A)** *Mt*Cut melting temperature curves at different CaCl_2_ concentrations in DSF experiments. Protein unfolding was monitored by detecting SYPRO orange fluorescence changes. **(B)** Comparison of cutinase and PET hydrolase activities in the absence/presence of different CaCl_2_ concentrations.

Enzyme thermostability and activity were enhanced by Ca^2+^; by adding 1 mM CaCl_2_, the optimum temperature (for *p*NP-C_4_ hydrolysis) of *Mt*Cut increased from 35 to 40°C, and also thermostability was considerably enhanced (Figures 1B,D). Notably, 60% activity was detected at 55°C upon Ca^2+^ addition, while only 30% activity was observed in the absence of Ca^2+^. Enzyme Ca^2+^-enhanced thermostability was confirmed by DSF, with the *T*_m_ determined as the minimal value of each melting peak (Figure 2A). The *T*_m_ value of *Mt*Cut was 33°C without Ca^2+^ but increased to 35, 39.5, and 41.5°C at Ca^2+^ concentrations of 10, 100, and 300 mM, respectively. *Mt*Cut activity towards *p*NP-C_4_ increased with increasing Ca^2+^concentration, while the *p*NP-C_4_ hydrolase activity was inhibited by 33% in the presence of 1 mM EDTA (Supplementary Figure S3). *Mt*Cut PET hydrolysis was also enhanced by Ca^2+^, as observed by *p*NP-C_4_ hydrolase activity, but was maximum at 10–100 mM CaCl_2_ and decreased at higher Ca^2+^ concentrations (Figure 2B). In addition, the optimum enzyme temperature for PET was 45°C (Supplementary Figure S4), which was higher than *p*NP-C_4_. Similar Ca^2+^-activating behaviors toward *p*NP-esters and PET hydrolysis were previously reported for another cutinase, Cut190 from *S. viridis* (Kawai et al., 2014).

### PET Hydrolysis by *Mt*Cut

**FIGURE 3:**
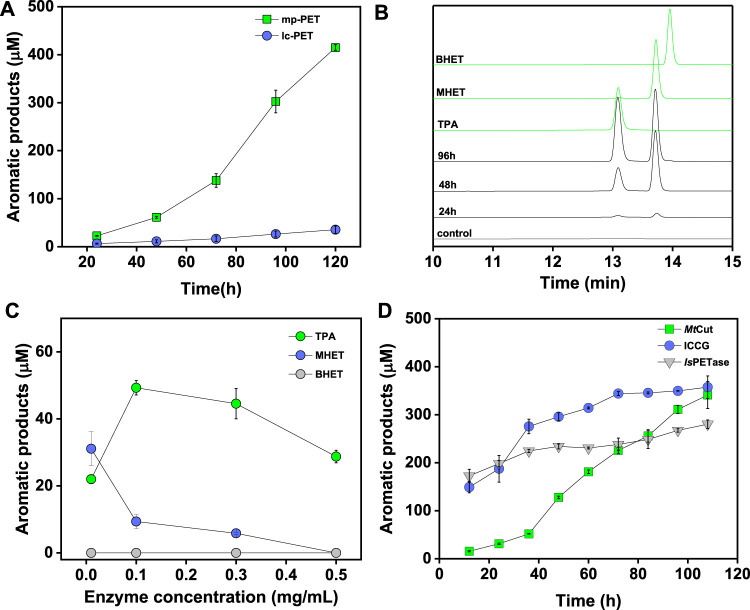
PET hydrolysis by *Mt*Cut. Released aromatic products were quantified as the sum of detected compounds (TPA, MHET, and BHET). **(A)** Progress curves showing variations in total aromatic product release over time toward micro-particle PET (mp-PET) and low crystallinity PET film (lc-PET). **(B)** High performance liquid chromatography spectrum of aromatic products released from mp-PET. **(C)** The effects of enzyme concentration on PET hydrolysis product profiles. **(D)** PET hydrolysis progresses comparisons for *Mt*Cut, *Is*PETase, and ICCG.

To analyze the PET-hydrolyzing activity of *Mt*Cut, microparticle (mp-PET) and lc-PET films with distinct morphology and crystallinity were selected, and hydrolytic performances were evaluated using released aromatic monomers (combined BHET, MHET, and TPA). Although the mp-PET possessed a higher crystallinity (42.67%) than the lc-PET films (10.32%), *Mt*Cut displayed more efficient degradation against mp-PET (Figure 3A), suggesting the PET hydrolysis was influenced more by surface-enzyme contact than crystallinity. Therefore, mp-PET was used as a standard substrate for further PET hydrolysis analyses. On closer inspection of released aromatic products, *Mt*Cut (10 μg/ml) hydrolyzed mp-PET to MHET and TPA, plus trace BHET amounts (Figure 3B). When the enzyme concentration was increased, the proportion of MHET decreased, with no MHET detected after a 500 μg/ml *Mt*Cut reaction over 72 h (Figure 3C). Thus, we speculated that *Mt*Cut exhibited higher hydrolytic activity against BHET than MHET, as confirmed by kinetic analyses on both compounds (Table 2). Although *Mt*Cut showed a higher affinity towards MHET (*K*_m_ = 0.75 mM) than BHET (*K*_m_ = 3.07 mM), the *k*_cat_ value for BHET (4.23 s^−1^) was much higher than MHET (0.13 s^−1^), leading to an overall 7-fold higher catalytic efficiency for BHET.

**TABLE 2:** Kinetic hydrolysis parameters of MHET and BHET by *Mt*Cut, ICCG, and *Is*PETase^a^.

Enzyme	Substrate	*K*_m_ (mM)	*k*_cat_ (s^−1^)	*k*_cat_/*K*_m_ (M^−1^s^−1^)
*Mt*Cut	MHET	0.75 ± 0.11	0.13 ± 0.01	(1.76 ± 0.14) × 10^2^
	BHET	3.07 ± 0.93	4.27 ± 1.15	(1.41 ± 0.07) × 10^3^
ICCG	MHET	0.43 ± 0.02	(3.20 ± 0.10) × 10^−3^	6.75 ± 0.32
	BHET	2.58 ± 0.98	2.29 ± 0.95	(1.00 ± 0.17) × 10^3^
*Is*PETase	MHET	0.72 ± 0.02	(1.39 ± 0.06) × 10^−2^	19.19 ± 0.45
	BHET	4.48 ± 0.54	5.95 ± 0.62	(1.33 ± 0.02) × 10^3^

a Kinetic assays were performed at 40°C in triplicate.

**FIGURE 4:**
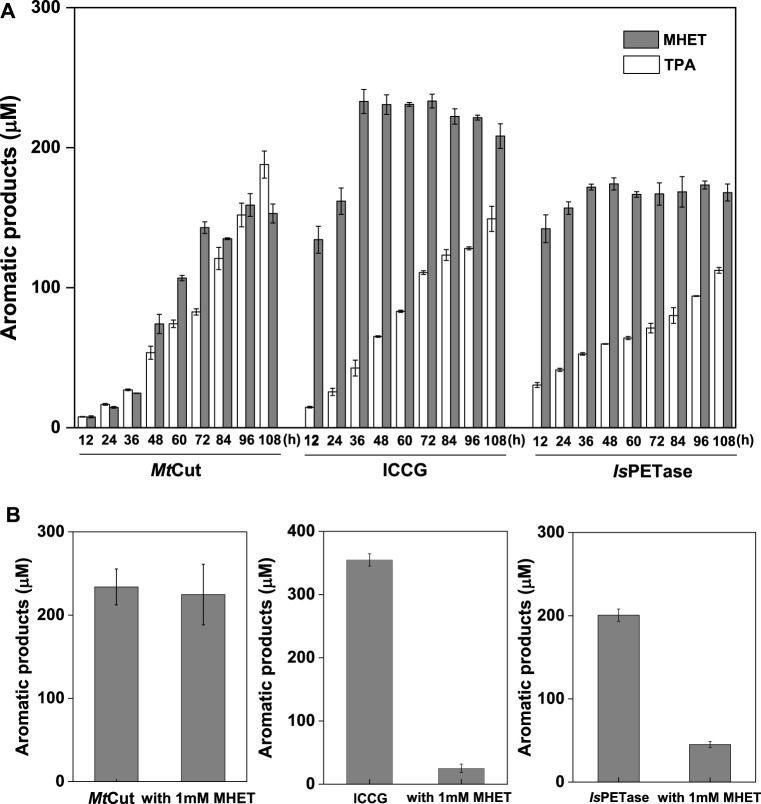
Product release and inhibition. **(A)** Product quantification of mp-PET hydrolysis by *Mt*Cut, ICCG, and *Is*PETase enzymes over 108 h at 40°C, with 2 mg/ml PET and 10 μg/ml enzyme. **(B)** The inhibitory effects of MHET at a final concentration of 1 mM on *Mt*Cut, ICCG, and *Is*PETase activities. PET hydrolysis products were analyzed in a 72 h reaction under standard conditions.

PET hydrolysis by *Mt*Cut at 40°C was compared with the well-characterized *Is*PETase and ICCG (variant of LCC with improved activity and thermostability) molecules. *Is*PETase and ICCG outperformed *Mt*Cut at the early stages in a 60 h-reaction in terms of total released aromatic compounds, whereas *Mt*Cut was better at later reaction stages (Figure 3D). Product profiles showed that the three enzymes not only differed in terms of reaction rates, but also in terms of product diversity. All enzymes generated MHET and TPA as main products. However, *Is*PETase and ICCG showed a higher MHET to TPA ratio, while *Mt*Cut released both products in equal quantities (Figure 4A). In terms of barely altered MHET levels at later stages and the relative slower rate of TPA production by *Is*PETase and ICCG, we speculated that MHET very likely inhibited the enzyme activity. When mp-PET hydrolysis reactions were performed in the presence of MHET, significant inhibitory effects were detected toward *Is*PETase and ICCG. In contrast, no MHET inhibition toward *Mt*Cut was observed (Figure 4B). In Addition, *Is*PETase and ICCG exhibited weak hydrolyzing activity against MHET at 40°C, while the catalytic efficiency of *Mt*Cut was 10-fold higher (Table 2). The catalytic efficiencies on MHET were found to be in accordance with binding energy by molecular docking analyses (Supplementary Figure S5). Thus, different MHET effects and activities generated distinct PET hydrolysis product profiles, and suggested *Mt*Cut was a novel PET hydrolase, uninhibited by MHET.

### Thermal Adaptation Mechanisms by MD Simulation

**FIGURE 5:**
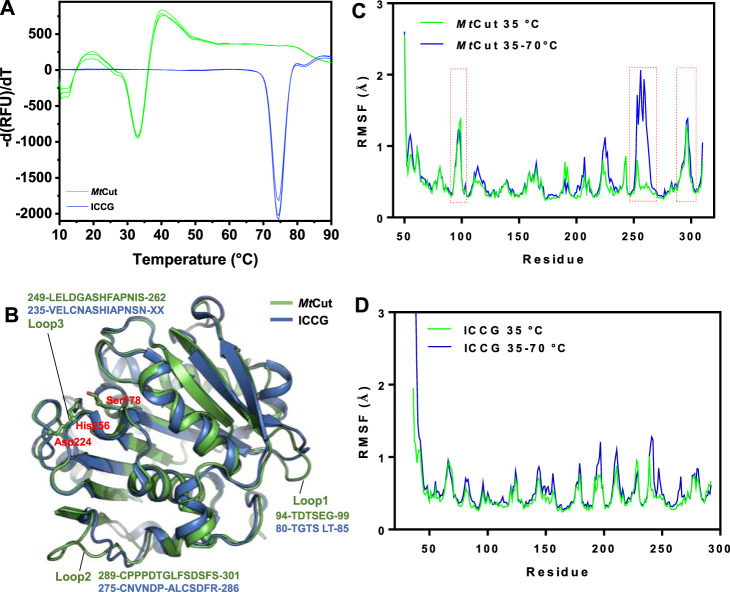
Structural features associated with temperature adaptation. **(A)** Comparing *Mt*Cut and ICCG melting temperature curves. **(B)** Structures of *Mt*Cut and ICCG (PDB entry: 6THT). The catalytic triad (Ser178, Asp224, and His256) is represented as a stick model. Root mean square fluctuations (RMSF) (angstroms) of C_α_ atoms of *Mt*Cut **(C)** and ICCG **(D)** structures at different temperatures. The 100 ns simulation trajectories at 35°C and heating from 35 to 70°C for each system were superposed and then used to calculate the RMSF.

Using DSF, *Mt*Cut thermostability was compared with ICCG, one of the most stable PET hydrolases, using DSF (Figure 5A). One obvious distinction was observed between *Mt*Cut and ICCG and related to temperature adaptation; *T*_m_ values were 33 and 74.5°C, respectively. To elucidate the temperature adaptation mechanism of both homologous enzymes and identify key components to engineer thermostability in cutinase-like enzymes, *Mt*Cut and ICCG (PDB entry: 6THT) structures were analyzed. Since our attempts to obtain *Mt*Cut crystals failed, we built an atomic coordinate structure using AlphaFold2 (Figure 5B). MD simulations at different temperatures were performed on *Mt*Cut and ICCG enzymes to investigate global structure changes and localized flexibility of individual amino acid residues.

When the system temperature increased from 35 to 70°C in the 100 ns simulation, the recorded root mean square deviation (RMSD) of *Mt*Cut was divided into two segments and that of ICCG changed from chaotic zone to stable zone (Supplementary Figures S6A,B). Our structure cluster analysis showed that *Mt*Cut transformed from a single-group to a multi-group (Supplementary Figure S6C), indicating the protein structure had rapidly changed. As expected, ICCG adjusted to a single group with increasing temperature (Supplementary Figure S6D). Both computational and experimental results for ICCG were in agreement and showed the enzyme was more stable under higher temperatures. Root mean square fluctuation (RMSF) analysis of each residue was used to describe structural flexibility during the heating program. When compared with ICCG, two loops (residues 94–99 and 289–301) on the *Mt*Cut surface exhibited higher RMSF values (Figures 5C,D). Interestingly, the latter loop was reportedly involved in Ca^2+^ binding, and the two mutations D238C/S283C (corresponding to residues Asp252 and Ser301 on *Mt*Cut) allowed disulfide-bond formation and thermal stabilization of LCC (Tournier et al., 2020). Moreover, the RMSF of *Mt*Cut residues 249–262, which comprised the catalytic loop with the active site His256, were remarkably increased when the simulation temperature increased from 35 to 70°C. While the paired region of thermophilic ICCG was resistant to heat denaturation, less RMSF increases were observed. Thus, we propose the catalytic loop which undergoes conformational changes in response to environmental temperature likely controls temperature adaptation in cutinase-like PET hydrolases.

## Discussion

Enzymes active against PET represent valuable scaffolds for plastic recycling applications and the elucidation of substrate recognition and catalysis mechanisms. The characterized enzymes involved in PET depolymerization are primarily cutinases belonging to the α/β hydrolase superfamily (Chen et al., 2013). In this study, we examined the cutinase activity of *Mt*Cut toward *p*NP esters and found the enzyme exhibited similar substrate selectivity and catalytic efficiency to reported cutinases (Thumarat et al., 2012; Arnling Baath et al., 2022) which are more active on *p*NP-C_6_ or C_8_ than C_2_ or long-chain fatty acid esters. *Mt*Cut also efficiently catalyzed PET depolymerization with a comparable activity similar to the well-studied *Is*PETase and ICCG enzymes at ambient temperatures (Figure 3D). PET hydrolysis by these cutinases was very likely due to a surface exposed active site in a shallow cleft that enabled enzymes to accommodate and hydrolyze insoluble aromatic polyesters (Figure 5B). In addition, surface pretreatment to increase the surface dimensions is essential for a more efficient enzymatic hydrolysis during PET biorecycling; *Mt*Cut displayed a higher activity on microparticles with higher crystallinity when compared with flat films with lower crystallinity (Figure 3A).

The main PET hydrolysis products from *Mt*Cut enzymatic reactions were species containing one aromatic ring (MHET and TPA), possibly indicating an exo-type hydrolyzing manner. The *Mt*Cut product profile agreed with previous PET hydrolysis catalyzed by other cutinases (Yoshida et al., 2016). In spite of this, cutinases appear to perform the scissions of PET polymer chains via diverse modes. For example, Thc_Cut1 and Thc_Cut2 hydrolyzed PET in an endo-type manner to generate PET oligomers as main products (Tournier et al., 2020; Arnling Baath et al., 2022), while TfCut2 performed PET degradation *via* a combinatorial exo- and endo-type mechanism (Wei et al., 2019). These observations corroborate the notion that α/β hydrolase-fold enzymes catalyze promiscuous mechanisms via the same serine-histidine-aspartate catalytic triad (Rauwerdink and Kazlauskas, 2015).

The enzymatic degradation of PET was influenced by product inhibition. As a major hydrolysis product, MHET strongly binds to TfCut2 and is slowly hydrolyzed (Barth et al., 2015a). The inhibition of PET hydrolysis by MHET was therefore identified as the main factor limiting the polyester hydrolase. To circumvent this bottleneck, one particular strategy involves a membrane filter reactor for the continuous removal of low molecular degradation products to reduce inhibition (Barth et al., 2015b). We observed that product inhibition was very likely ubiquitous among cutinases, as other well-characterized PET hydrolases, *Is*PETase and ICCG, are strongly inhibited by MHET (Figure 4B). Interestingly, PET hydrolysis by *Mt*Cut was not inhibited by MHET, suggesting this enzyme was superior to other and aforementioned PET hydrolases. Based on our kinetic analyses, *Mt*Cut displayed a 10-fold higher hydrolytic efficiency against MHET than *Is*PETase and ICCG (Table 2), suggesting released MHET was rapidly degraded. In considering the similar substrate affinities (*K*_m_ values) of the other PET hydrolases against MHET, we speculate the higher activity of *Mt*Cut may be due to a more structural flexibility of the active site (Figures 5C,D).

While thermostable PET hydrolases promote efficient PET depolymerization, considering the *T*_g_ values, some studies performed PET hydrolysis at ambient temperatures to conserve energy (Sagong et al., 2021). Therefore, temperature adaptation mechanisms of PET hydrolases are generating a good deal of interest with respect to the potential applications in areas such as biorecycling and waste treatment. Since the effects of Ca^2+^ are crucial for the activity and stability of *Mt*Cut, we identified the conserved residues, Ser301 and Asp252 of *Mt*Cut, as the putative calcium binding sites by structural alignment with Est119 (Supplementary Figure S7). Substitution of either site with alanine decreased the optimum temperature and thermal stability of *Mt*Cut (Figures 1B,D). The calcium activating manners of mutant D252A and S301A also differed from wild type (Figure 2B). Additionally, those two residues were substituted by cysteine to introduce disulfide bond at the corresponding sites, and D252CS301C displayed more efficient hydrolysis on PET than wild type *Mt*Cut at higher temperatures of 45 and 50°C (Supplementary Figure S4). These results supported the regulatory function of Ca^2+^ binding on enzyme activity and stability. Large conformational changes in several loop regions upon Ca^2+^ binding were observed on cutinase Cut190 (Kawai et al., 2014), and the structural dynamics were reported to be essential for the PET hydrolysis at higher temperatures depending on the presence of Ca^2+^ (Numoto et al., 2018). The protein *Mt*Cut is homologous to Cut190 and Est119 in terms of both structure and function, we assume that similar mechanisms of Ca^2+^ based activation and stabilization may occur in *Mt*Cut. Due to a remarkable *T*_m_ difference of 40°C between *Mt*Cut and ICCG, the two homologous enzymes are type models to investigate the thermal adaptation mechanism of PET hydrolases. From structural and computational analyse, the cold adaptation of *Mt*Cut may be attributed to flexible features within the protein structure. Specifically, three surface-exposed loops on *Mt*Cut exhibited distinct differences with ICCG in terms of conformational dynamics, which could regulate the catalysis and temperature adaptation of bacterial cutinases. Further studies, reengineering these enzyme regions to generate conformational dynamics for mutants with better thermostability, are warranted.

## Conclusion

We characterized a novel cutinase-type PET-degrading enzyme, *Mt*Cut, which exhibited efficient PET-hydrolyzing activity at the ambient temperatures. Biochemical studies showed *Mt*Cut performed PET hydrolysis in an exo-type manner, with both enzyme activity and thermal stability improved by calcium ions addition. Importantly, *Mt*Cut was not inhibited by the hydrolysis product, MHET, suggesting *Mt*Cut is a better enzyme than other PET hydrolases. Moreover, the structural elements responsible for regulating thermal adaptation were identified by computational analyses. Taken together, this novel enzyme provides insights on PET degradation and temperature adaptation mechanisms of cutinase-type enzymes, and suggests a promising *in vitro* platform to generate better performing enzymes for PET biorecycling.

## Data Availability

The original contributions presented in the study are included in the article/Supplementary Material, further inquiries can be directed to the corresponding authors.
