# Supplementary material for: Catalytic Features and Thermal Adaptation Mechanisms of a Deep Sea Bacterial Cutinase-Type Poly(Ethylene Terephthalate) Hydrolase
Source: Front Bioeng Biotechnol. 2022 Apr 26;10:865787. doi: 10.3389/fbioe.2022.865787 (PMC9086285; doi:10.3389/fbioe.2022.865787)
Supplement: Supplementary file 1 [file DataSheet1.docx]

**Catalytic features and thermal adaptation mechanisms of a deep sea bacterial cutinase-type poly(ethylene terephthalate) hydrolase**

**(Supplementary information)**

Yu Liu^a,d^, Chen Liu ^c^, Huan Liu^a,d^, Qi Zeng^a,d^, Xinpeng Tian^a,b,d^, Lijuan Long^a,b,d*^, Jian Yang^a,b,d*^

^a^CAS Key Laboratory of Tropical Marine Bio-resources and Ecology, Guangdong Key Laboratory of Marine Materia Medica, South China Sea Institute of Oceanology, Chinese Academy of Sciences, Guangzhou 510301, China

^b^Southern Marine Science and Engineering Guangdong Laboratory (Guangzhou), Guangzhou 511458, China

^c^Guangzhou Quality Supervision and Testing Institute, Guangzhou 511447, China

^d^University of the Chinese Academy of Sciences, Beijing 100049, People’s Republic of China

^*^Corresponding author: Lijuan Long (email: longlj@scsio.ac.cn); Jian Yang (email: yangjian@scsio.ac.cn)

**Figure S1** Amino acid sequence alignment of *Mt*Cut with previously described PET hydrolases, including Cut190 from *Saccharomonospora viridis*, Est119 from *Thermobifida alba*, TfCut1 from *Thermobifida fusca*, LCC from leaf-branch uncultured bacterium, and *Is*PETase from *Ideonella sakaiensis*. The catalytic triad is indicated by red stars; the conserved pentapeptide sequence motif among cutinases are indicated by green frame; the loop structures proposed to be associated with thermal adaptation are indicated by blue frames.

**Figure S2** Molecular docking analysis of *p*NP-esters against *Mt*Cut. (a) Correlation between catalytic efficiency and binding energies of *p*NP-C_2_ to C_18_. The linear regression is calculated for the *p*NP-ester substrates, the slope of this curve is -0.8 and the *R*^2^ is 0.9. (b) The binding modes between *p*NP-esters and *Mt*Cut.

**Figure S3** Inhibitory effect of 1 mM EDTA on *p*NP-C4 hydrolase activity of *Mt*Cut.

**Figure S4** Temperature dependency of PET hydrolysis by *Mt*Cut and its variant D252CS301C.

**Figure S5** Molecular docking analysis of MHET and BHET against *Mt*Cut.

**Figure S6** RMSD and cluster analysis of *Mt*Cut and ICCG during the 100 ns-simulation of the heating process. (a) RMSD of the backbone of *Mt*Cut during 100 ns heating process. (b) RMSD of the backbone of ICCG. Cluster analysis of *Mt*Cut (c) and ICCG (d) during 100 ns heating simulations.

**Figure S7** Addition of a disulfide bridge to improve *Mt*Cut thermostability. Disulfide bonds of *Thermobifida alba* Est119 (PDB ID 3WYN) and divalent metal ions are shown as green, the mutation sites of *Mt*Cut are shown as purple sticks.

Table S1 Primers used for site-directed mutagenesis

| Primer | Oligonucleotide sequence(5’-3’) |
| --- | --- |
| D252C-F | GCCTACCTGGAGCTGTGTGGCGCGAGCCACTTC |
| D252C-R | ACACAGCTCCAGGTAGGCGCGTTCCAGGTCCTCGT |
| S301C-F | CTCTTCTCGGACTTCTGTGACTACCGGGACTCC |
| S301C-R | ACAGAAGTCCGAGAAGAGCCCGGTGTCGGG |
| D252A-F | GCCTACCTGGAGCTGGCAGGCGCGAGCCACTTC |
| D252A-R | TGCCAGCTCCAGGTAGGCGCGTTCCAGGTCCTCGT |
| S301A-F | CTCTTCTCGGACTTCGCAGACTACCGGGACTCC |
| S301A-R | TGCGAAGTCCGAGAAGAGCCCGGTGTCGGG |
